# Cross-sectional study using hepatic steatosis detected on ultrasound and its correlation with metabolic syndrome

**DOI:** 10.6026/973206300212918

**Published:** 2025-08-31

**Authors:** Sneha Subhash Mahalpure, Vishwak Sena M.D., Chinmay Anilkumar Keshwani, Mohammed Shihas

**Affiliations:** 1 Department of Anatomy, N.K.P Salve Institute of Medical Sciences & Research Centre and Lata Mangeshkar Hospital, Nagpur, Maharashtra, India; 2 Department of Medical Gastroenterology, Sri Ramachandra Institute of Higher Education and Research, Tamil Nadu, India; 3 Department of Emergency Medicine, East Lancashire NHS Trust, Lancashire County, United Kingdom; 4 Department of Medicine, Aster Hospitals Dubai, Dubai, UAE

**Keywords:** Hepatic steatosis, fatty liver, ultrasound, metabolic syndrome, cross-sectional study, insulin resistance, abdominal imaging

## Abstract

Hepatic steatosis, frequently identified incidentally using ultrasound, is increasingly linked with metabolic syndrome. This cross-sectional study assessed 132 patients undergoing abdominal ultrasound to detect fatty liver and its association with metabolic risk factors. A significant correlation was found between hepatic steatosis and components such as central obesity, elevated triglycerides and insulin resistance. Thus, we show underscore the role of routine imaging in identifying individuals at risk. Early detection can guide timely interventions to prevent long-term complications.

## Background:

Metabolic dysfunction-associated fatty liver disease (MAFLD), originally known as non-alcoholic fatty liver disease (NAFLD), has practical and straightforward diagnostic criteria that are superior to NAFLD for determining individuals at elevated risk for liver fibrosis and extrahepatic manifestations, including chronic kidney disease (CKD), type 2 diabetes mellitus (T2DM), and cardiovascular disease (CVD) [1]. Non-viral non-alcoholic fatty liver disease (NAFLD) is an increasingly recognized condition that accompanies an increase in obesity. NAFLD is known to be associated with various metabolic abnormalities including central obesity, type 2 diabetes, dyslipidemia, and high blood pressure, which are also well-established cardiovascular risk factors [2]. Hepatic steatosis, commonly referred to as fatty liver, is a condition characterized by the accumulation of fat in hepatocytes, typically exceeding 5% of liver weight [3]. With the rising prevalence of obesity, sedentary lifestyle and unhealthy dietary habits, non-alcoholic fatty liver disease (NAFLD) has emerged as the most common chronic liver condition worldwide [4]. NAFLD encompasses a spectrum of liver abnormalities ranging from simple steatosis to non-alcoholic steatohepatitis (NASH), which can further progress to cirrhosis and hepatocellular carcinoma [5]. Ultrasound remains the most widely used imaging modality for the initial detection of hepatic steatosis due to its non-invasive nature, accessibility and cost-effectiveness [6]. Incidental findings of fatty liver on routine sonography often prompt further clinical evaluation, especially in asymptomatic individuals [7]. Metabolic syndrome is a cluster of conditions-including central obesity, dyslipidemia, hypertension and impaired glucose tolerance that significantly increase the risk of cardiovascular disease and type 2 diabetes mellitus [8]. A growing body of evidence suggests a strong association between hepatic steatosis and metabolic syndrome, with both conditions often coexisting and sharing similar pathophysiological mechanisms, such as insulin resistance and chronic inflammation [9]. Therefore, it is of interest to explore the prevalence of hepatic steatosis detected on ultrasound and evaluate its correlation with the presence of metabolic syndrome in a cross-sectional cohort of adult patients.

## Materials and Methods:

This cross-sectional study was conducted in the radiology and internal medicine departments of a tertiary care hospital over a period of six months. A total of 132 adult patients aged between 25 and 70 years who underwent abdominal ultrasonography for various clinical indications were included consecutively. Patients with known chronic liver diseases, significant alcohol intake (defined as >20g/day for women and >30g/day for men), hepatotoxic drug use, or viral hepatitis were excluded to eliminate confounding factors. Ultrasound examinations were performed using a high-resolution B-mode ultrasound machine with a 3.5-5 MHz convex transducer. Hepatic steatosis was diagnosed based on standard sonographic criteria such as increased hepatic echogenicity compared to renal cortex, blurring of vascular margins and posterior beam attenuation. The degree of steatosis was graded qualitatively as mild, moderate, or severe. Clinical and anthropometric data were collected at the time of imaging, including body mass index (BMI), waist circumference and blood pressure. Fasting blood samples were obtained to measure serum triglycerides, HDL cholesterol and fasting plasma glucose. Metabolic syndrome was diagnosed based on the revised National Cholesterol Education Program Adult Treatment Panel III (NCEP ATP III) criteria, which require the presence of at least three of the following: increased waist circumference, elevated triglycerides (≥150 mg/dL), reduced HDL cholesterol (<40 mg/dL in men, <50 mg/dL in women), elevated blood pressure (≥130/85 mmHg) and elevated fasting glucose (≥100 mg/dL). Data were compiled and statistically analyzed to assess the correlation between ultrasound-detected hepatic steatosis and the presence of metabolic syndrome and its individual components.

## Results:

Among 132 patients, hepatic steatosis was detected in 78 individuals (59.1%) on ultrasound. A strong association was observed between hepatic steatosis and metabolic syndrome, with significant correlations noted with central obesity, elevated triglycerides and impaired fasting glucose. The following tables detail the distribution of hepatic steatosis, its grading and its correlation with various metabolic parameters. Table 1 (see PDF) shows this table displays the demographic distribution of the study participants, revealing a higher prevalence of steatosis among males and individuals in the 41-60 age groups. Table 2 (see PDF) shows the majority of patients with hepatic steatosis had a BMI ≥25 kg/m^2^, indicating a strong association with overweight and obesity. Table 3 (see PDF) shows waist circumference, a marker of central obesity, was significantly higher among those with hepatic steatosis. Table 4 (see PDF) shows raised triglyceride levels were predominantly seen in patients with hepatic steatosis. Table 5 (see PDF) shows Low HDL levels were more frequent in the steatosis group, aligning with known metabolic risk profiles. Table 6 (see PDF) shows elevated fasting plasma glucose was significantly associated with hepatic steatosis. Table 7 (see PDF) shows a substantial portion of patients with steatosis met 3 or more criteria for metabolic syndrome. Table 8 (see PDF) shows grading of hepatic steatosis on ultrasound showed most patients had moderate to severe fatty infiltration. Table 9 (see PDF) shows Patients with higher steatosis grades had a higher prevalence of metabolic syndrome. Table 10 (see PDF) shows the overall prevalence of metabolic syndrome was significantly higher in patients with steatosis compared to those without.

## Discussion:

The findings of this cross-sectional study emphasize the significant association between ultrasound-detected hepatic steatosis and metabolic syndrome. With a prevalence of 59.1%, hepatic steatosis was frequently encountered in the studied population, reflecting global trends of increasing non-alcoholic fatty liver disease (NAFLD), particularly in urban and semi-urban healthcare settings. The predominance of steatosis among individuals aged 41-60 years and males align with known epidemiological patterns [10]. One of the most striking observations was the high co-occurrence of hepatic steatosis with multiple components of metabolic syndrome. Central obesity, as measured by waist circumference, was the most common metabolic derangement, followed by hypertriglyceridemia and impaired fasting glucose. These parameters are not only diagnostic markers of metabolic syndrome but also contribute to the pathophysiological development of hepatic fat accumulation through mechanisms involving insulin resistance, lipotoxicity and chronic low-grade inflammation [11]. The gradation of steatosis on ultrasound revealed that moderate to severe grades were predominant and a linear trend was observed with the number of metabolic syndrome criteria met. This dose-dependent relationship suggests that hepatic fat accumulation is both a marker and a mediator of systemic metabolic dysfunction. In particular, 100% of individuals with Grade 3 steatosis had metabolic syndrome, underlining the liver's central role in metabolic homeostasis [12]. The strong positive correlation between hepatic steatosis and metabolic markers, particularly BMI, triglycerides and fasting glucose, reinforces the concept of NAFLD being the hepatic manifestation of metabolic syndrome. The inverse relationship with HDL levels further corroborates existing literature linking dyslipidemia with fatty liver disease. These results are consistent with prior studies that have established hepatic steatosis as an independent risk factor for cardiovascular morbidity and type 2 diabetes [13]. Ultrasonography proved to be a valuable, non-invasive tool in screening for hepatic steatosis and indirectly identifying patients at high risk for metabolic syndrome. Given its accessibility and cost-effectiveness, routine ultrasound evaluation especially in individuals with obesity, diabetes, or dyslipidemia may serve as an early warning system for impending metabolic derangement. However, certain limitations should be acknowledged [14]. The cross-sectional nature of the study precludes the establishment of temporal or causal relationships. Ultrasound, though effective, is operator-dependent and less sensitive in detecting mild steatosis compared to advanced imaging techniques. Additionally, the study did not account for dietary habits, physical activity levels, or genetic predispositions, which could influence both hepatic fat accumulation and metabolic profiles [15]. This study strengthens the evidence for a strong link between hepatic steatosis detected on ultrasound and metabolic syndrome. It advocates for the integration of hepatic imaging into metabolic risk assessments, which may help in identifying high-risk individuals and implementing early lifestyle or pharmacologic interventions.

## Conclusion:

Ultrasound-detected hepatic steatosis is significantly associated with metabolic syndrome and its components, particularly central obesity, hypertriglyceridemia and insulin resistance. The liver acts as a central organ reflecting systemic metabolic health. Early identification of steatosis via ultrasound can aid in timely intervention and risk reduction for cardiovascular and diabetic complications.
